# Thyroid dysfunction and risk of atrial fibrillation in patients with hypertrophic obstructive cardiomyopathy: a multicenter cohort study

**DOI:** 10.3389/fendo.2026.1801303

**Published:** 2026-04-17

**Authors:** Weijie Zhang, Fushi Piao, Wei Fu, Zhouxu Geng, Mengshen Wang, Yuhan He, Zongyang Li, Honghou He, Zhuchang Tian, Shengsong Zhu, Qiaonan Ye, Min Lin, Zhiyuan Zhang, Jing Chen

**Affiliations:** 1 Department of Emergency, Fuwai Central China Cardiovascular Hospital, Zhengzhou, Henan, China; 2 Department of Cardiology, Peking University Shenzhen Hospital, Shenzhen, Guangdong, China; 3 Department of Cardiology, The First Hospital of Hebei Medical University; Graduate School of Hebei Medical University, Shijiazhuang, Hebei, China; 4 Department of Computer Science and Engineering, The Hong Kong University of Science and Technology, Hong Kong, China; 5 Department of Cardiology, State Key Laboratory of Cardiovascular Disease, Fuwai Hospital, National Center for Cardiovascular Diseases, Chinese Academy of Medical Sciences and Peking Union Medical College, Beijing, China; 6 Department of Plastic and Burn Surgery, National Key Clinical Construction Specialty, The Affiliated Hospital of Southwest Medical University, Luzhou, Sichuan, China; 7 Division of Cardiac Arrhythmia, Cardiac and Vascular Center, The University of Hong Kong–Shenzhen Hospital, Shenzhen, Guangdong, China

**Keywords:** atrial fibrillation, hypertrophic obstructive cardiomyopathy, risk factors, thyroid dysfunction, thyroid-stimulating hormone

## Abstract

**Background:**

Atrial fibrillation (AF) is a common and clinically important arrhythmia in patients with hypertrophic obstructive cardiomyopathy (HOCM). Thyroid dysfunction influences atrial electrophysiology and remodeling; however, its association with AF in HOCM remains poorly defined.

**Methods:**

We retrospectively analyzed 755 consecutive patients with HOCM (mean age 48.65 ± 14.12 years; 60.0% male) from three tertiary centers (2019–2024). Patients were categorized by thyroid-stimulating hormone (TSH) levels as <0.55, 0.55–2.49 (reference), 2.50–9.99, and ≥10.00 mIU/L. Multivariable logistic regression and restricted cubic spline analyses were performed. Supplementary Cox proportional hazards regression was performed as a sensitivity analysis using exact event timing.

**Results:**

During 12-month follow-up, 107 patients developed incident AF (cumulative incidence, 14.2%). After multivariable adjustment, elevated TSH was independently associated with AF (TSH 2.50–9.99 mIU/L: OR 1.981, 95% CI 1.126–3.512; TSH ≥10.00 mIU/L: OR 4.306, 95% CI 1.061–17.485). Supplementary Cox regression yielded consistent results (TSH 2.50–9.99 mIU/L: HR 1.824, 95% CI 1.087–3.061; TSH ≥10.00 mIU/L: HR 3.685, 95% CI 1.018–13.337). Restricted cubic spline analysis demonstrated a U-shaped association between TSH and AF risk.

**Conclusions:**

In this multicenter retrospective cohort of patients with HOCM, both subclinical and overt hypothyroidism were associated with a higher risk of incident AF. Our findings indicate that thyroid function status may be associated with AF risk and could potentially contribute to risk stratification; prospective interventional studies are warranted to determine whether thyroid optimization can reduce AF risk.

## Introduction

Hypertrophic cardiomyopathy (HCM) is among the most common inherited cardiac diseases, affecting approximately 1 in 500 individuals in the general population (1). A substantial proportion of patients develop left ventricular outflow tract obstruction, termed hypertrophic obstructive cardiomyopathy (HOCM), which is associated with exertional dyspnea, angina, syncope, ventricular arrhythmias, and an increased risk of sudden cardiac death (2, 3). Atrial fibrillation (AF) is the most frequent sustained arrhythmia in HCM, occurring in up to 20–30% of patients over their lifetime (4). AF carries particularly important consequences in HOCM because atrial contraction is critical for ventricular filling in the setting of diastolic dysfunction and a noncompliant hypertrophied ventricle (5). Clinically, AF in HOCM is associated with functional deterioration, higher hospitalization rates, and a markedly increased risk of ischemic stroke (6–8). Accordingly, current guidelines recommend anticoagulation for all patients with HCM and AF regardless of conventional stroke risk scores (4, 9).

Thyroid hormones exert broad effects on cardiovascular physiology, including heart rate, myocardial contractility, vascular tone, and cardiac electrophysiology (10). Overt hyperthyroidism is a well-established trigger for tachyarrhythmias, particularly AF, through shortening of atrial refractoriness and increased triggered activity (11). Subclinical hyperthyroidism is also associated with a substantially higher risk of AF in population-based cohorts (12, 13). In contrast, hypothyroidism is linked to impaired diastolic relaxation, increased vascular stiffness, and myocardial fibrosis, which may promote atrial remodeling and arrhythmogenesis (14). Consistent with these mechanisms, epidemiologic studies suggest a nonlinear, U-shaped association between thyroid-stimulating hormone (TSH) levels and AF risk across the spectrum of thyroid function (15, 16).

In cardiomyopathy and heart failure populations, thyroid dysfunction may further aggravate hemodynamic stress and myocardial remodeling. In HCM, reduced free triiodothyronine (FT3) levels have been associated with adverse clinical outcomes, potentially reflecting impaired peripheral conversion of thyroxine (T4) to triiodothyronine (T3) and more advanced disease status (17–19). However, whether thyroid dysfunction—particularly elevated TSH consistent with subclinical or overt hypothyroidism—predicts incident AF in AF-free patients with HOCM remains insufficiently characterized.

We therefore conducted a multicenter retrospective cohort study of 755 AF-free patients with HOCM to evaluate the association between thyroid function and the risk of incident AF during follow-up. We hypothesized that both subclinical and overt hypothyroidism would be independently associated with a higher odds of incident AF after adjustment for established risk determinants, including age, left atrial size, and NT-proBNP.

## Methods

### Study design and population

This multicenter retrospective cohort study was conducted at three tertiary referral centers in China (Henan Provincial People’s Hospital, The First Hospital of Hebei Medical University, and The University of Hong Kong–Shenzhen Hospital). Consecutive patients evaluated for HOCM between January 2019 and December 2024 were screened. The study was approved by the Ethics Committee of all three institutions. Written informed consent was obtained from all patients or their legal guardians/next of kin in accordance with the national legislation and institutional requirements.

HOCM was diagnosed according to contemporary guideline criteria as hypertrophic cardiomyopathy with left ventricular outflow tract (LVOT) obstruction. Hypertrophic cardiomyopathy was defined as otherwise unexplained left ventricular hypertrophy (maximal wall thickness ≥15 mm, or ≥13 mm when supported by additional clinical evidence) in the absence of abnormal loading conditions. LVOT obstruction was defined as a resting or provoked LVOT gradient ≥30 mmHg.

For the primary analysis, participants were required to have baseline thyroid function measurements and no documented AF at baseline. Patients with baseline AF, prior thyroid surgery, overt thyroid hormone replacement or antithyroid therapy before baseline, or missing 12-month outcome ascertainment were excluded.

### Clinical and echocardiographic assessment

Baseline demographic and clinical data were extracted from electronic medical records, including age, sex, body mass index, comorbidities, NYHA functional class, history of syncope, and medication use. Laboratory parameters included NT-proBNP, serum creatinine, fasting glucose, and lipid profiles.

Transthoracic echocardiography was performed according to standardized protocols. Key echocardiographic parameters included left atrial (LA) anteroposterior diameter, interventricular septal thickness, LVOT gradient, and left ventricular systolic function. LA diameter was analyzed as a continuous variable as a surrogate of atrial remodeling. We acknowledge that LA volume index would provide a more comprehensive assessment of atrial size; however, LA anteroposterior diameter was the only standardized measurement consistently available across all three participating centers.

### Laboratory measurements and thyroid function categories

Baseline thyroid function testing included TSH, FT3, and FT4. Thyroid indices were measured using an electrochemiluminescence immunoassay (Roche Diagnostics). For primary analyses, patients were categorized into four predefined TSH strata: <0.55, 0.55–2.49, 2.50–9.99, and ≥10.00 mIU/L. The 0.55–2.49 mIU/L category served as the reference group.

### Outcome definition and follow-up

The primary outcome was incident AF within 12 months after baseline among patients without prior AF. The fixed 12-month endpoint was selected to provide a standardized, uniform follow-up period across all three centers, minimizing differential ascertainment bias, and to capture early-onset AF events most likely related to baseline thyroid status rather than intervening clinical events. AF was defined as AF lasting ≥30 seconds documented by 12-lead ECG or 24-hour Holter monitoring, or a clinician-confirmed diagnosis with supporting ECG evidence.

AF ascertainment was performed through systematic rhythm evaluation at the 12-month follow-up visit and review of interim outpatient visits, hospital admissions, and ECG/Holter records. The date of first documentation of AF within 12 months was recorded as the event date.

### Statistical analysis

The association between baseline thyroid function and 12-month incident AF was evaluated using logistic regression, with results reported as odds ratios (ORs) and 95% confidence intervals (CIs). Because the follow-up duration was fixed at 12 months for all participants and censoring was minimal, logistic regression provides an appropriate estimate of risk within a defined time window; in such settings, odds ratios approximate relative risks when event incidence is moderate. Multivariable models were prespecified based on clinical relevance and prior literature, while maintaining an appropriate events-per-variable ratio to minimize the risk of overfitting. Multicollinearity was assessed using variance inflation factors. Restricted cubic spline functions were fitted to explore potential nonlinear associations.

As a prespecified sensitivity analysis, Cox proportional hazards regression was performed using the exact timing of AF events to account for time-to-event information. The proportional hazards assumption was assessed using Schoenfeld residuals. Results were reported as hazard ratios (HRs) with 95% CIs.

All analyses were conducted using R software (version 4.3.0). A two-sided P value <0.05 was considered statistically significant.

## Results

### Baseline characteristics

A total of 755 patients with HOCM were included (mean age 48.65 ± 14.12 years; 60.0% male). Patients were categorized into four groups based on baseline TSH levels: <0.55 mIU/L (n=78), 0.55–2.49 mIU/L (reference, n=536), 2.50–9.99 mIU/L (n=121), and ≥10.00 mIU/L (n=20). Age, FT3, FT4 and NT-proBNP levels differed significantly across TSH categories (P < 0.001). Other baseline variables were broadly comparable (all P > 0.05) (**Table 1**).

**Table 1:** Baseline clinical characteristics of the study population.

Variable	<0.55(n=78)	0.55–2.49(n=536)	2.50–9.99(n=121)	≥10(n=20)	*P* value
Age, years (mean ± SD)	48.51 ± 14.52	49.46 ± 13.62	51.54 ± 14.81	54.67 ± 12.16	<0.001
Male, n (%)	45 (60.0%)	335 (61.7%)	73 (59.7%)	13(65.0%)	0.687
Body mass index, kg/m² (mean ± SD)	24.55 ± 3.82	24.91 ± 3.56	24.75 ± 3.61	24.84 ± 3.56	0.352
Hypertension, n (%)	27 (35.0%)	203 (38.5%)	39 (30.6%)	7 (36.0)	0.062
Diabetes, n (%)	10 (12.1%)	78 (15.0%)	14 (11.6%)	3 (11.6%)	0.215
Dyslipidemia, n (%)	18 (25.0%)	146 (27.1%)	31 (24.7%)	5 (25.0)	0.641
Coronary artery disease, n (%)	7 (9.0%)	57 (11.2%)	10 (8.6%)	2 (10.0)	0.295
Prior stroke/TIA, n (%)	3 (4.1%)	30 (5.6%)	5 (3.9%)	1 (5.0%)	0.227
NYHA class III-IV, n (%)	15 (17.7%)	151 (28.0%)	19 (16.0%)	3 (15.0%)	0.32
β-blocker use, n (%)	53 (68.1%)	383 (71.0%)	82 (67.6%)	14 (70.0%)	0.538
Calcium channel blocker use, n (%)	18 (22.0%)	123 (23.4%)	25 (21.8%)	4 (20.0%)	0.81
Interventricular septal thickness, mm (mean ± SD)	19.02 ± 4.03	19.55 ± 4.21	18.97 ± 4.02	18.81 ± 4.14	0.452
LVOT gradient at rest, mmHg (mean ± SD)	42.16 ± 19.52	43.26 ± 20.01	41.06 ± 19.42	42.57 ± 18.84	0.156
Left atrial diameter, mm (mean ± SD)	40.56 ± 7.22	42.11 ± 7.05	39.83 ± 6.87	40.12 ± 6.86	0.485
LVEF, % (mean ± SD)	66.72 ± 7.14	66.12 ± 8.01	65.86 ± 7.55	66.57 ± 7.06	0.372
NT-proBNP, pg/mL (median [IQR])	960.50 [440.13-2102.82]	1101.5 [901.12-3205.55]	893.26 [415.52-1920.85]	925.71 [421.82-2011.81]	<0.001
Serum creatinine, µmol/L (mean ± SD)	82.51 ± 24.01	92.43 ± 28.45	81.54 ± 23.21	85.77 ± 24.86	0.689
FT3, pmol/L (mean ± SD)	4.51 ± 1.36	4.25 ± 1.20	4.19 ± 1.28	4.15 ± 1.31	<0.001
FT4, ng/dL (mean ± SD)	1.76 ± 0.27	1.65 ±0.29	1.63 ± 0.28	1.60 ± 0.31	<0.001

### Baseline characteristics stratified by incident AF

Overall, patients who developed AF were older (54.29 ± 13.14 vs 47.91 ± 14.09 years, P < 0.001) and had higher NYHA class, larger LA diameters (45.23 ± 7.24 vs 39.54 ± 6.75 mm, P < 0.001), and higher NT-proBNP levels. Patients with AF also had higher serum creatinine and TSH, and lower FT3 levels (all P<0.05) (**Table 2**).

**Table 2:** Baseline characteristics of the study population stratified by incident atrial fibrillation status.

Variable	Overall (N=755)	AF (n=107)	Non-AF (n=648)	*P* value
Age, years (mean ± SD)	48.18 ± 14.94	54.29 ± 13.14	47.91 ± 14.09	<0.001
Male, n (%)	453 (60.0)	66 (61.7)	387 (59.7)	0.682
Body mass index, kg/m² (mean ± SD)	24.61 ± 3.62	24.85 ± 3.72	24.56 ± 3.61	0.351
Hypertension, n (%)	241 (31.9)	43 (40.2)	198 (30.6)	0.063
Diabetes, n (%)	91 (12.1)	16 (15.0)	75 (11.6)	0.214
Dyslipidemia, n (%)	189 (25.0)	29 (27.1)	160 (24.7)	0.647
Coronary artery disease, n (%)	68 (9.0)	12 (11.2)	56 (8.6)	0.296
Prior stroke/TIA, n (%)	31 (4.1)	6 (5.6)	25 (3.9)	0.222
NYHA class III-IV, n (%)	134 (17.7)	30 (28.0)	104 (16.0)	0.003
β-blocker use, n (%)	514 (68.1)	76 (71.0)	438 (67.6)	0.531
Calcium channel blocker use, n (%)	166 (22.0)	25 (23.4)	141 (21.8)	0.814
Antiarrhythmic drug use, n (%)	70 (9.3)	13 (12.1)	57 (8.8)	0.282
Anticoagulant use, n (%)	119 (15.8)	41 (38.3)	78 (12.0)	<0.001
Interventricular septal thickness, mm (mean ± SD)	19.03 ± 4.06	19.51 ± 4.27	18.92 ± 4.05	0.121
LVOT gradient at rest, mmHg (mean ± SD)	42.23 ± 19.16	43.10 ± 20.02	41.98 ± 19.69	0.347
Left atrial diameter, mm (mean ± SD)	40.61 ± 7.12	45.23 ± 7.24	39.54 ± 6.75	<0.001
LVEF, % (mean ± SD)	66.14 ± 7.21	65.45 ± 8.65	66.78 ± 7.13	0.206
NT-proBNP, pg/mL (median [IQR])	960.26 [440.51-2100.27]	1800.74 [900.13-3200.86]	890.45 [410.62-1900.27]	<0.001
Serum creatinine, µmol/L (mean ± SD)	82.22± 24.17	92.51 ± 28.56	80.14 ± 23.72	0.004
FT3, pmol/L (mean ± SD)	4.51 ± 0.86	4.20 ± 0.90	4.55 ± 0.85	0.012
FT4, ng/dL (mean ± SD)	1.65 ± 0.32	1.76 ± 0.35	1.63 ± 0.31	0.008
TSH, mIU/L (median [IQR])	2.14 [1.22-3.86]	2.62 [1.17-5.91]	2.06 [1.27-3.44]	0.003

LVOT, left ventricular outflow tract; LVEF, left ventricular ejection fraction; NT-proBNP, N-terminal pro-B-type natriuretic peptide; FT3, free triiodothyronine; FT4, free thyroxine; TSH, thyroid-stimulating hormone. AF, atrial fibrillation; NYHA, New York Heart Association; TIA, transient ischemic attack.

### Incidence of AF by TSH categories

**Figure 1:**
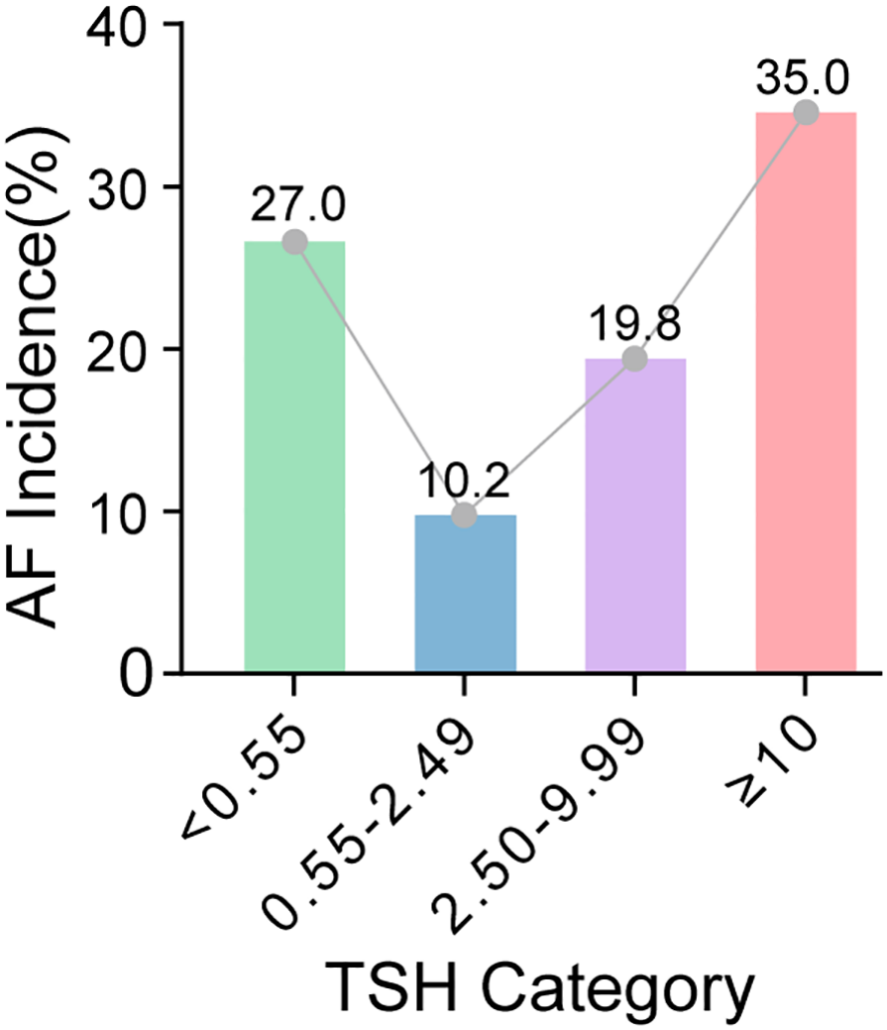
Incidence of atrial fibrillation by baseline TSH categories in patients with HOCM. Bars represent the proportion of patients who developed AF during follow-up within each baseline TSH stratum (<0.55, 0.55–2.49, 2.50–9.99, and ≥10.00 mIU/L). Values above the bars indicate the incidence (%) in each category. The connecting line depicts the trend in AF incidence across increasing TSH categories.

Cumulative incidence of AF varied significantly across TSH categories (**Figure 1**; **Table 3**). The highest incidence was in patients with TSH ≥10.00 mIU/L (35.0%), followed by TSH 2.50–9.99 mIU/L (19.8%). The reference group had 10.2% incidence, while TSH <0.55 mIU/L had 27.0%.

**Table 3:** One-year incidence of atrial fibrillation stratified by baseline TSH categories.

TSH category (mIU/L)	Patients, n	AF cases, n	Incident AF events, %	Non-AF, n
<0.55	78	21	26.9	57
0.55-2.49 (reference)	536	55	10.3	481
2.50-9.99	121	24	19.8	97
≥10.00	20	7	35	13
**Total**	**755**	**107**	**14.2**	**648**

Overall comparison: χ² test for trend, p < 0.001.

Bold values in the Total row indicate the overall values for the entire study cohort.

### Multivariable analysis of TSH and AF risk

**Figure 2:**
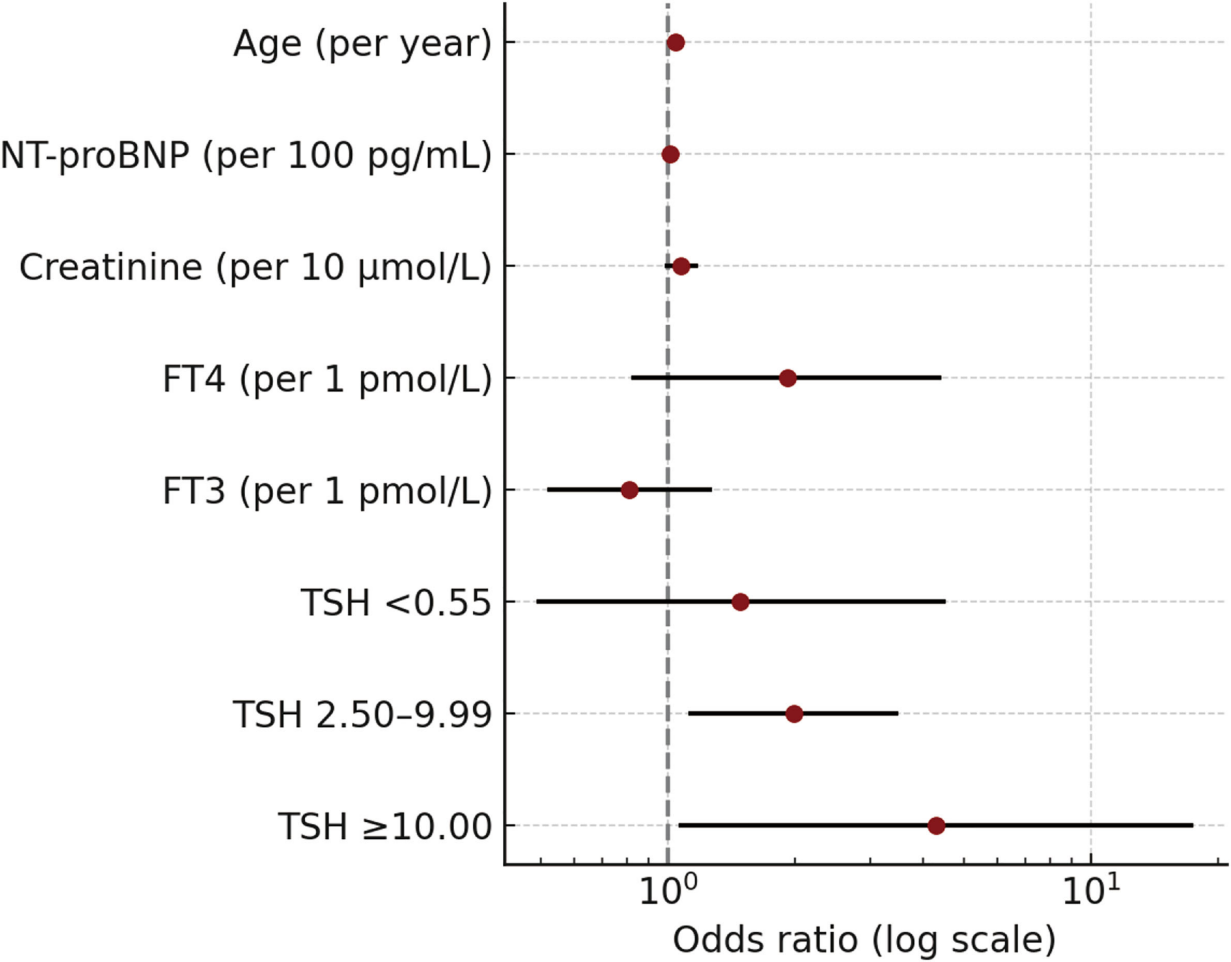
Multivariable-adjusted odds ratios for atrial fibrillation in HOCM patients. Multivariable-adjusted odds ratios for atrial fibrillation in patients with hypertrophic obstructive cardiomyopathy are shown. Odds ratios were derived from a logistic regression model adjusted for age, sex, NT-proBNP, serum creatinine, left atrial diameter, free triiodothyronine (FT3), free thyroxine (FT4), and TSH categories. CI, confidence interval; NT-proBNP, N-terminal pro-B-type natriuretic peptide.

Multivariable logistic regression revealed that elevated TSH was independently associated with increased AF risk: TSH 2.50–9.99 mIU/L (aOR 1.981, 95% CI 1.126–3.512, P = 0.020) and TSH ≥10.00 mIU/L (aOR 4.306, 95% CI 1.061–17.485, P = 0.041) (**Table 4**; **Figure 2**).

**Table 4:** Logistic regression for predictors of atrial fibrillation.

Variable	Model	OR (95% CI)	*P* value
Age, per 1-year increase	Univariate	1.045 (1.027-1.063)	<0.001
	Multivariable	1.040 (1.022-1.059)	<0.001
NT-proBNP, per 100 pg/mL	Univariate	1.017 (1.006-1.029)	0.003
	Multivariable	1.010 (1.003-1.020)	0.006
Serum creatinine, per 10 µmol/L	Univariate	1.131 (1.036-1.244)	0.006
	Multivariable	1.072 (0.982-1.184)	0.131
FT4, per 1 ng/dL	Univariate	3.345 (1.489-7.507)	0.004
	Multivariable	1.912 (0.822-4.435)	0.135
FT3, per 1 pmol/L	Univariate	0.625 (0.398-0.972)	0.035
	Multivariable	0.814 (0.524-1.279)	0.362
TSH <0.55 vs 0.55-2.49	Univariate	1.732 (0.715-4.216)	0.236
	Multivariable	1.486 (0.492-4.525)	0.497
TSH 2.50-9.99 vs 0.55-2.49	Univariate	2.172 (1.286-3.671)	0.004
	Multivariable	1.981 (1.126-3.512)	0.02
TSH ≥10.00 vs 0.55-2.49	Univariate	4.462 (1.184-16.921)	0.028
	Multivariable	4.306 (1.061-17.485)	0.041

### Sensitivity analysis using cox proportional hazards regression

Supplementary Cox proportional hazards regression, utilizing the exact timing of AF events, yielded results consistent with the primary analysis. The proportional hazards assumption was tested using Schoenfeld residuals and was not violated (global test P = 0.312). Compared with the reference group, patients with TSH 2.50–9.99 mIU/L had HR 1.824 (95% CI 1.087–3.061, P = 0.023), and those with TSH ≥10.00 mIU/L had HR 3.685 (95% CI 1.018–13.337, P = 0.047). Patients with TSH <0.55 mIU/L showed a non-significant trend (HR 1.418, 95% CI 0.512–3.928, P = 0.502). The concordance between approaches supports the robustness of the associations (**Supplementary Table 1**).

### Nonlinear association between TSH and AF risk

**Figure 3:**
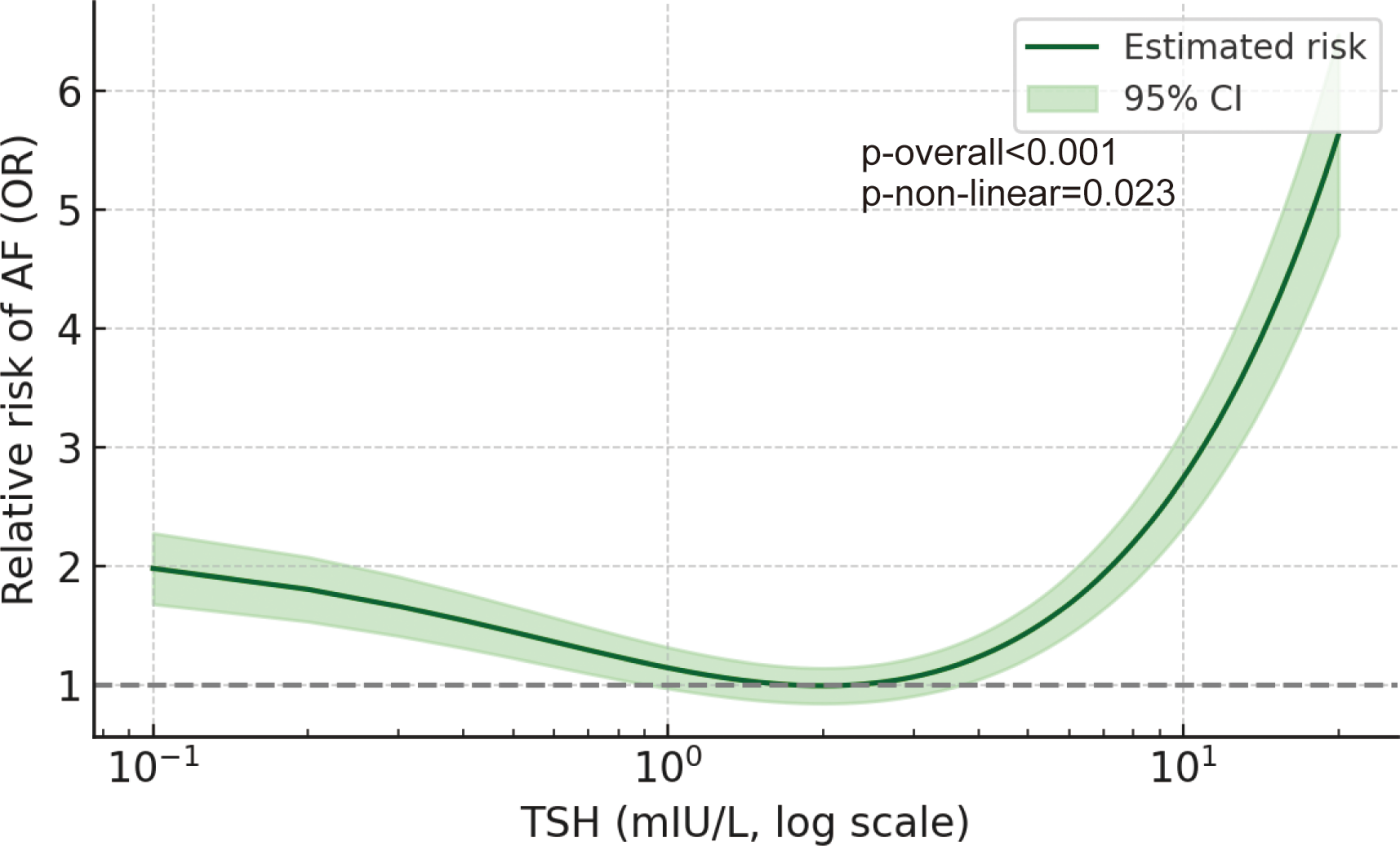
Restricted cubic spline analysis of TSH and AF risk. Restricted cubic spline analysis showing the nonlinear association between thyroid-stimulating hormone levels and the odds of atrial fibrillation in patients with hypertrophic obstructive cardiomyopathy. The solid line represents the adjusted odds ratio, and the shaded area indicates the 95% confidence interval. AF, atrial fibrillation; TSH, thyroid-stimulating hormone.

Restricted cubic spline analysis revealed a nonlinear, U-shaped association between TSH and AF odds (**Figure 3**). The lowest risk was at TSH approximately 1–3 mIU/L, while both low and high TSH were associated with increased AF risk.

## Discussion

This multicenter retrospective study demonstrates a significant and nonlinear association between thyroid dysfunction and atrial fibrillation in patients with HOCM. Both subclinical hypothyroidism (TSH 2.50–9.99 mIU/L) and overt hypothyroidism (TSH ≥10.00 mIU/L) were independently associated with higher odds of incident AF, while suppressed TSH showed a nonsignificant but consistent trend. These associations persisted after adjustment for age, NT-proBNP, and left atrial size. Supplementary Cox proportional hazards regression, which accounts for exact event timing, confirmed these findings and demonstrated consistent effect estimates.

Our findings are consistent with prior population-based studies reporting a U-shaped association between thyroid function and AF risk (20). Meta-analyses have demonstrated that both elevated and suppressed TSH levels are associated with incident AF (15, 16). The present study extends these observations to a high-risk population with structural heart disease and severe diastolic dysfunction.

Several pathophysiological mechanisms may underlie the observed association. Excess thyroid hormone shortens atrial refractory periods, increases β-adrenergic sensitivity, and enhances triggered activity (21–23). Conversely, hypothyroidism has been associated with interstitial myocardial fibrosis, endothelial dysfunction, and impaired diastolic relaxation (24). In patients with HOCM, these adverse effects may be further magnified.

An additional mechanistic link may involve alterations in peripheral thyroid hormone metabolism. Patients with AF exhibited lower FT3 levels, consistent with prior reports (18, 25). Impaired conversion of thyroxine to triiodothyronine may contribute to maladaptive myocardial remodeling (26–28). Both high and low extremes of thyroid function may exert deleterious effects on atrial structure and electrophysiology in HOCM (29).

From a clinical perspective, our findings suggest that routine thyroid function assessment in HOCM patients may provide additional information for AF risk stratification. Although current guidelines do not specifically address thyroid function (30), our data suggest thyroid assessment may be a useful component of comprehensive HOCM evaluation. However, it is important to emphasize that our observational data cannot establish whether correction of thyroid dysfunction would reduce AF incidence, and prospective interventional studies are needed.

This study has several strengths, including a relatively large multicenter cohort, standardized echocardiographic characterization, complementary categorical and spline-based analyses, and concordance between logistic and Cox regression approaches.

Nonetheless, several limitations merit consideration. First, the retrospective design precludes causal inference, and residual confounding from unmeasured variables cannot be excluded. Our multivariable model focused on key AF predictors while maintaining an appropriate events-per-variable ratio given 107 outcome events. Important unmeasured confounders include left atrial volume index, left atrial strain, genetic subtype, alcohol intake, myocardial fibrosis extent, obstructive severity dynamics, septal reduction therapy, and antiarrhythmic drug changes during follow-up. Echocardiographic interobserver variability was not formally assessed.

Second, AF ascertainment relied on routine 12-month ECG, 24-hour Holter, and clinical records. Without continuous monitoring (e.g., implantable loop recorders), paroxysmal AF may have been missed. The CRYSTAL-AF trial showed prolonged monitoring detected AF in 12.4% vs 2.0% by conventional methods (31), and the ASSERT study demonstrated subclinical atrial tachyarrhythmias were far more prevalent than clinically recognized (32). This limitation would bias results toward the null, suggesting our estimates may be conservative.

Third, thyroid function was assessed at a single baseline time point. A single TSH measurement may not reflect long-term thyroid status given intra-individual variability from non-thyroidal illness, medications, and hemodynamic changes. Longitudinal thyroid data were not available.

Fourth, reverse causality cannot be entirely excluded. Although baseline AF was rigorously excluded, subclinical hemodynamic alterations could theoretically influence thyroid hormone levels prior to AF onset.

Fifth, limited sample sizes in extreme TSH categories—particularly TSH ≥10.00 mIU/L (n=20) and TSH <0.55 mIU/L (n=78)—result in wide confidence intervals and reduced statistical power. These effect estimates should be interpreted with caution.

Sixth, the 12-month follow-up may underestimate cumulative AF incidence, as AF in HOCM is progressive. Longer follow-up may yield different effect estimates.

Collectively, prospective studies with longer follow-up, continuous rhythm monitoring, serial thyroid assessments, and comprehensive covariate adjustment are warranted to confirm our findings and determine whether targeted thyroid management can mitigate AF risk in HOCM.

## Conclusion

In this multicenter retrospective cohort of patients with hypertrophic obstructive cardiomyopathy, thyroid dysfunction was independently and nonlinearly associated with atrial fibrillation. Relative to the reference TSH range, both subclinical and overt hypothyroidism were linked to higher adjusted odds of AF. Our findings indicate that thyroid function status may be associated with AF risk and could potentially contribute to risk stratification in patients with HOCM. These findings support systematic thyroid function assessment as part of comprehensive HOCM care and warrant prospective interventional studies to determine whether optimizing thyroid status can reduce AF risk and downstream clinical burden.

## Data Availability

The data analyzed in this study is subject to the following licenses/restrictions: The dataset used in this study is based on retrospective clinical data, which is subject to ethical restrictions and privacy protection regulations. As such, access to the dataset is restricted to authorized personnel involved in the research, and data cannot be shared publicly due to patient confidentiality agreements. Requests for access to the dataset will be considered on a case-by-case basis and must comply with institutional review board (IRB) approval and applicable data protection laws. Data access will be provided under controlled conditions, and only anonymized data will be shared for further research purposes. Requests to access these datasets should be directed to Zhiyuan Zhang, yuanzhizhang296@163.com.
