# Supplementary material for: Thyroid dysfunction and risk of atrial fibrillation in patients with hypertrophic obstructive cardiomyopathy: a multicenter cohort study
Source: Front Endocrinol (Lausanne). 2026 Apr 17;17:1801303. doi: 10.3389/fendo.2026.1801303 (PMC13132738; doi:10.3389/fendo.2026.1801303)
Supplement: Supplementary file 1 [file Table1.docx]

**Supplementary Table S1. Cox proportional hazards regression for predictors of atrial fibrillation**

| **Variable** | **Model** | **HR (95% CI)** | **p value** | **PH test p** |
| --- | --- | --- | --- | --- |
| Age, per 1-year increase | Univariate | 1.038 (1.022–1.054) | <0.001 |  |
|  | Multivariable | 1.033 (1.016–1.051) | <0.001 | 0.451 |
| NT-proBNP, per 100 pg/mL | Univariate | 1.014 (1.005–1.024) | 0.004 |  |
|  | Multivariable | 1.009 (1.002–1.017) | 0.008 | 0.523 |
| Serum creatinine, per 10 µmol/L | Univariate | 1.11 (1.02–1.21) | 0.008 |  |
|  | Multivariable | 1.06 (0.97–1.16) | 0.15 | 0.387 |
| FT4, per 1 pmol/L | Univariate | 2.89 (1.32–6.34) | 0.008 |  |
|  | Multivariable | 1.74 (0.76–3.98) | 0.19 | 0.614 |
| FT3, per 1 pmol/L | Univariate | 0.65 (0.41–1.02) | 0.062 |  |
|  | Multivariable | 0.83 (0.54–1.28) | 0.40 | 0.291 |
| TSH <0.55 vs 0.55–2.49 | Univariate | 1.56 (0.58–4.18) | 0.38 |  |
|  | Multivariable | 1.418 (0.512–3.928) | 0.502 | 0.718 |
| TSH 2.50–9.99 vs 0.55–2.49 | Univariate | 1.98 (1.21–3.24) | 0.006 |  |
|  | Multivariable | 1.824 (1.087–3.061) | 0.023 | 0.356 |
| TSH ≥10.00 vs 0.55–2.49 | Univariate | 3.92 (1.12–13.72) | 0.033 |  |
|  | Multivariable | 3.685 (1.018–13.337) | 0.047 | 0.482 |

Model specification: Multivariable model included age, sex, NT-proBNP, serum creatinine, left atrial diameter, FT3, FT4, and TSH category; variance inflation factors were <2 for all covariates.

Global Schoenfeld residuals test: P = 0.312, confirming proportional hazards assumption was not violated.

Abbreviations: HR, hazard ratio; CI, confidence interval; PH, proportional hazards; NT-proBNP, N-terminal pro-B-type natriuretic peptide; FT3, free triiodothyronine; FT4, free thyroxine; TSH, thyroid-stimulating hormone.
